# The insecticide resistance status of malaria vectors in the Mekong region

**DOI:** 10.1186/1475-2875-7-102

**Published:** 2008-06-05

**Authors:** Wim Van Bortel, Ho Dinh Trung, Le Khanh Thuan, Tho Sochantha, Duong Socheat, Chalao Sumrandee, Visut Baimai, Kalouna Keokenchanh, Phompida Samlane, Patricia Roelants, Leen Denis, Katrijn Verhaeghen, Valerie Obsomer, Marc Coosemans

**Affiliations:** 1 Institute of Tropical Medicine, Dept. Parasitology, Nationalestraat 155, B-2000 Antwerpen, Belgium; 2 National Institute of Malariology, Parasitology and Entomology, Dept. Entomology, Luong The Vinh street, B.C. 10.200 Tu Liem, Hanoi, Vietnam; 3 National Center for Malaria Control, Parasitology and Entomology, Dept. Entomology, 372 Monivong Boulevard, Phnom Penh, Cambodia; 4 Mahidol University, Dept Biology, Rama IV road, Bangkok 10400, Thailand; 5 Center of Malariology, Parasitology and Entomology, Dept. Entomology, Kualuang Road, Vientiane, Laos; 6 Université Catholique de Louvain, Dept. Of Environmental Sciences and Land Use Planning, Croix du sud 2/16, B-1348 Louvain-la-Neuve, Belgium; 7 Department of Biomedical Sciences, Faculty of Pharmaceutical, Veterinary and Biomedical Sciences, University of Antwerp, Universiteitsplein 1, B-2610 Antwerpen (Wilrijk), Belgium

## Abstract

**Background:**

Knowledge on insecticide resistance in target species is a basic requirement to guide insecticide use in malaria control programmes. Malaria transmission in the Mekong region is mainly concentrated in forested areas along the country borders, so that decisions on insecticide use should ideally be made at regional level. Consequently, cross-country monitoring of insecticide resistance is indispensable to acquire comparable baseline data on insecticide resistance.

**Methods:**

A network for the monitoring of insecticide resistance, MALVECASIA, was set up in the Mekong region in order to assess the insecticide resistance status of the major malaria vectors in Cambodia, Laos, Thailand, and Vietnam. From 2003 till 2005, bioassays were performed on adult mosquitoes using the standard WHO susceptibility test with diagnostic concentrations of permethrin 0.75% and DDT 4%. Additional tests were done with pyrethroid insecticides applied by the different national malaria control programmes.

**Results:**

*Anopheles dirus s.s*., the main vector in forested malaria foci, was susceptible to permethrin. However, in central Vietnam, it showed possible resistance to type II pyrethroids. In the Mekong delta, *Anopheles epiroticus *was highly resistant to all pyrethroid insecticides tested. It was susceptible to DDT, except near Ho Chi Minh City where it showed possible DDT resistance. In Vietnam, pyrethroid susceptible and tolerant *Anopheles minimus s.l*. populations were found, whereas *An. minimus s.l*. from Cambodia, Laos and Thailand were susceptible. Only two *An. minimus s.l*. populations showed DDT tolerance. *Anopheles vagus *was found resistant to DDT and to several pyrethroids in Vietnam and Cambodia.

**Conclusion:**

This is the first large scale, cross-country survey of insecticide resistance in *Anopheles *species in the Mekong Region. A unique baseline data on insecticide resistance for the Mekong region is now available, which enables the follow-up of trends in susceptibility status in the region and which will serve as the basis for further resistance management. Large differences in insecticide resistance status were observed among species and countries. In Vietnam, insecticide resistance was mainly observed in low or transmission-free areas, hence an immediate change of malaria vector control strategy is not required. Though, resistance management is important because the risk of migration of mosquitoes carrying resistance genes from non-endemic to endemic areas. Moreover, trends in resistance status should be carefully monitored and the impact of existing vector control tools on resistant populations should be assessed.

## Background

The important economic and social implications caused by malaria in the Mekong region have prompted governments to make this disease a public health priority and to implement integrated national malaria control programmes adapted to the specific needs of their individual countries [1-4]. The implementation of these comprehensive control programmes resulted in a significant reduction of malaria in the Mekong region. However, malaria is still an important disease in foci located in forested areas and along the country borders, from where it can spread to areas which are currently malaria free [5]. Vector control has played an essential role in the reduction of malaria in the Mekong region and is still indispensable to control malaria in endemic foci. Moreover, reducing transmission intensity is likely to slow the spread of drug resistance [6]. The available vector control methods rely on the use of insecticides for bed net impregnation (ITNs) or indoor spraying. Consequently, the development of insecticide resistance may jeopardize the vector control efforts. Hence, knowledge of vector resistance and changing trends of resistance in target species are basic requirements to guide insecticide use in malaria control programmes [7].

In Vietnam, insecticide resistance monitoring of *Anopheles *mosquitoes has been carried out regularly. Before 1989, DDT resistance has been found in the malaria vector *Anopheles epiroticus *(former *Anopheles sundaicus *[8]). From 1990 till 2000, pyrethroid resistance has been monitored in Vietnam showing that almost all tested species were susceptible except some populations of *Anopheles vagus *and *Anopheles minimus s.l*. [9,10]. In Thailand no evidence of insecticide resistance in malaria vectors was present before 1985 [11]. Between 1990 and 1997, DDT resistance has been detected in *Anopheles dirus s.l*. and *An. minimus s.l*. and permethrin resistance was found in a population of *An. minimus s.l*. from northern Thailand, based on a discriminative dosage of 0.25% permethrin [11]. However, the discriminative dosage for permethrin has been increased to 0.75% making historical comparison difficult [12]. Moreover, the existing information on the resistance status of the main malaria vectors, *An. dirus s.l*., *An. minimus s.l*. and *An. epiroticus*, in the Mekong region is patchy and region-wide comparable resistance data are needed to funnel the correct use of insecticides in malaria vector control. Therefore, a cross-country insecticide resistance monitoring network, MALVECASIA, was set-up, including Cambodia, Laos, Thailand and Vietnam, to define the insecticide susceptibility status of the major malaria vectors in different physio-geographical regions. This paper reports the results of the three years monitoring of insecticide resistance using the WHO-bioassays.

## Methods

### Mosquito sampling, identification and bioassays

From 2003 until 2005, adult mosquitoes were collected by different collection methods (indoor and outdoor human landing collection, collection on cattle and morning resting collections inside houses) in Cambodia, Laos, Thailand and Vietnam. Mosquitoes were identified morphologically in the field by use of a standardized key for medically important anophelines of south-east Asia [13]. *Anopheles dirus s.l., An. epiroticus*, *An. minimus s.l*. and *An. vagus *were subjected to the standard WHO bioassay the morning after the night collection. The first three test species are the main targets of the vector control programmes in the Mekong region. *Anopheles vagus*, which is a potential vector in south-east Asia, was included as indicator species. Larvae of this species are found in a large variety of sun-exposed breeding sites such as small pools, hoof prints, puddles, ditches often containing foul water and rice fields [14-16]. They are often found in the vicinity of human dwellings and are likely to be exposed to insecticides from agriculture activities.

Bioassays were performed on adult mosquitoes using the standard WHO susceptibility test with diagnostic concentrations of permethrin 0.75% and DDT 4% [12]. Additional tests were done with insecticides applied by the different national malaria control programmes: lambda-cyhalothrin 0.05% and deltamethrin 0.05% in Cambodia, alpha-cypermethrin 0.082% (30 mg/m^2^) and deltamethrin 0.05% in Laos, deltamethrin 0.05% in Thailand, and alpha-cypermethrin 0.082% (30 mg/m^2^), lambda-cyhalothrin 0.05% and deltamethrin 0.05% in Vietnam. Nowadays only pyrethroids are being applied in malaria vector control, but DDT was tested because it has been intensively used for vector control in the past. Moreover, it can be used for exploring cross-resistance with other insecticides, such as pyrethroids. All control and insecticide-impregnated papers were supplied by the Vector Control Research Unit, Universiti Sains Malaysia and were not used more than five times. Mosquitoes were exposed for 60 minutes in tubes places in vertical position. During exposure the number of mosquitoes knocked down was recorded after 10, 15, 20, 30, 40, 50 and 60 min. After exposure, mosquitoes were kept under observation for 24 h, supplied with 10% sugar solution and mortality was read after this 24 h period.

Adults could not always be collected in appropriate numbers (minimum 80) during one night so that replicates were tested over different days. Each day a control was tested alongside the exposure tubes. The bioassay result was corrected using the Abbott formula when the control mortality was between 5 and 20% [12]. Results were excluded from further analysis when the mortality in the controls exceeded 20%. A weighted mean was applied to summarize the mortality over different consecutive days. Weights were proportional to the number of specimens tested per day. The bioassay results were summarized in three resistance classes as defined by WHO [12]: (1) susceptible when mortality was 98% or higher, (2) possible resistant when mortality was between 97 and 80%, and (3) resistant when the mortality was lower than 80%.

Where possible bioassays were repeated in a different season or years (details can be found in the additional file). Larvae of *An. epiroticus *and *An. minimus *were collected in sites where resistance was detected and reared to adults to confirm the results of bioassays done on mosquitoes from the adult collection methods. Only a limited number of sites could be confirmed but the number of tested mosquitoes exceeded always 100.

### Molecular identification

All mosquitoes surviving the exposure to insecticide during the bioassay and at least an equal number of mosquitoes killed in the bioassay were subjected to molecular identification in order to identify the members of the different species complexes (*An. dirus s.l*. and *An. minimus s.l*.), and to verify the quality of the morphological identification. One to six legs per mosquito were used for genomic DNA extraction, applying the procedure described in Collins *et al *[17]. DNA was re-suspended in 25 μl TE buffer (10 mM Tris-HCl pH 8; 1 mM EDTA). Morphologically identified *An. minimus s.l*. mosquitoes were analysed by using a slightly adapted version of the PCR-RFLP developed by Van Bortel *et al *[18]. The restriction enzyme BsiZi was replaced by its isoschizomer Cfr13I. The reaction mixture contained 17 μl sterile water, 2.5 μl Tango™ buffer (provided by manufacturer), 0.5 μl of Cfr13I (10 U/μl) (Fermentas, St-Leon Rot, Germany) and 5 μl of the PCR product. The mixture was incubated for 2 h at 37°C. After incubation, the specimens were electrophoresed on a 3% mixed agarose gel (1.5% agarose and 1.5% small fragment agarose) and visualized under UV light after ethidium bromide staining. The same PCR-RFLP gave a different restriction pattern for *An. vagus *which could be used to verify the morphological identification of this species. The PCR-RFLP assay was adapted to enable the molecular identification of *An. epiroticus*. The restriction enzyme MwoI was selected on the basis of the ITS2 sequence of the original described *An. epiroticus *AY469855 [8] and used to confirm the morphological identification. *Anopheles dirus *mosquitoes were subjected to the multiplex PCR developed by Walton *et al *[19].

## Results

### Quality control of the morphological identification

Problems with the morphological identification were observed in Cambodia and Thailand (Table 1). In Cambodia, *An. minimus s.l*. was mainly confused with *Anopheles aconitus*, whereas in Thailand it was mainly confounded with *Anopheles maculatus*. Large differences in morphological misidentifications existed between the study sites which could be attributed to skills of the different field teams. The morphological identification improved towards the end of the project.

**Table 1:** The percentage of correct morphologically identified mosquitoes assessed by molecular identification assays.

Morphological identified *Anopheles *species	Percentage of correct identification by country			
	
	Cambodia	Laos	Thailand	Vietnam
*Anopheles dirus *complex	92 (466)	100 (104)	100 (183)	100 (520)
*Anopheles epiroticus*	94 (87)	Not present	87 (124)	97 (893)
*Anopheles minimus *complex	61 (458)	90 (336)	68 (133)	96 (2687)
*Anopheles vagus*	87 (1513)	99 (297)	Not collected	100 (1713)

Number of tested specimens between brackets.

The error rate was compared between killed and surviving mosquitoes in order to assess whether the misidentifications would influence the bioassay results. By study site no difference in identification error rate was seen between the dead and alive mosquitoes, except for *An. vagus *from the study site KTRA Cambodia (Fisher exact, p = 0.04). The misidentification did not influence the interpretation of the bioassays since the resistance classes as defined by WHO were not different when calculations were based on morphological identification alone or when different identification error rates among dead and live mosquitoes were taken into account.

### Bioassay results

**Figure 1:**
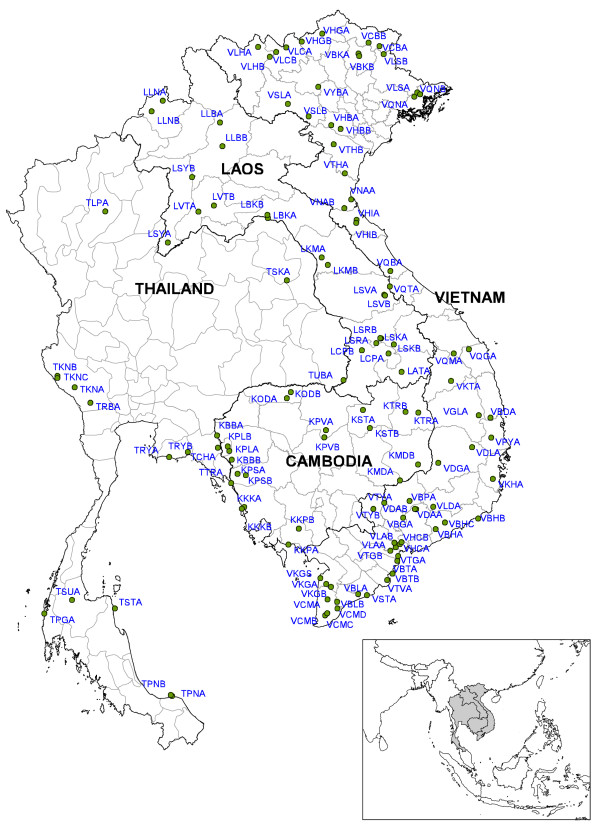
The MALVECASIA study sites with their corresponding codes.

**Figure 2:**
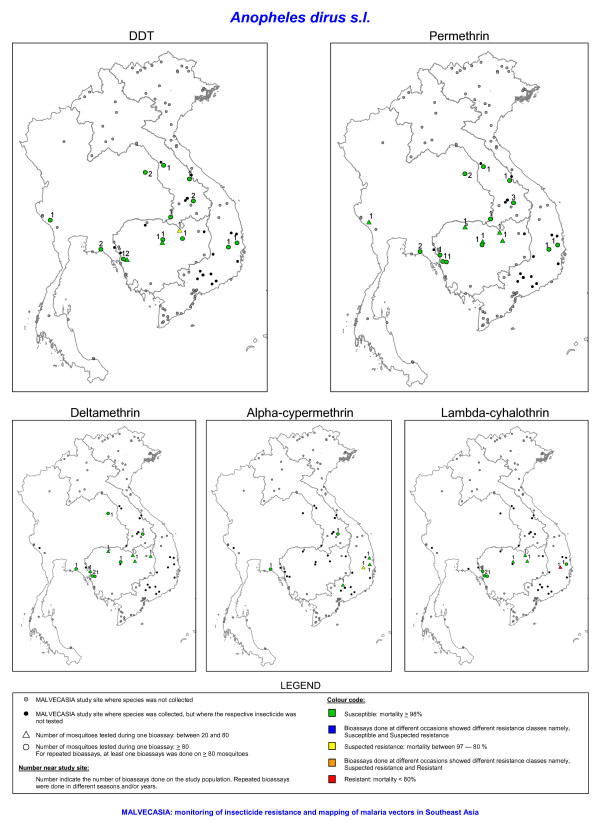
**The insecticide resistance status as defined by the mortality rate obtained with bioassays of *Anopheles dirus s.l*. for five insecticides**. The number of tested mosquitoes has been corrected for morphological misidentifications.

**Figure 3:**
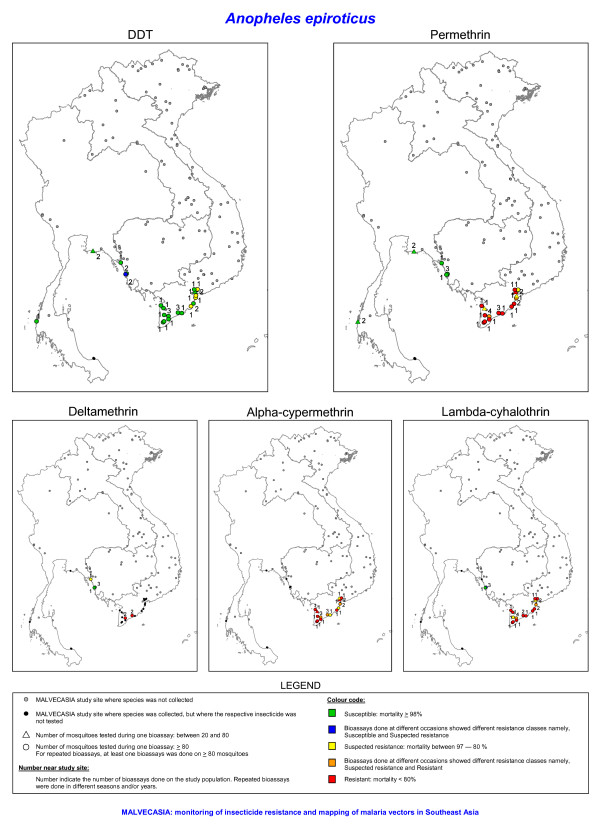
**The insecticide resistance status as defined by the mortality rate obtained with bioassays of *Anopheles epiroticus *for five insecticides**. The number of tested mosquitoes has been corrected for morphological misidentifications.

**Figure 4:**
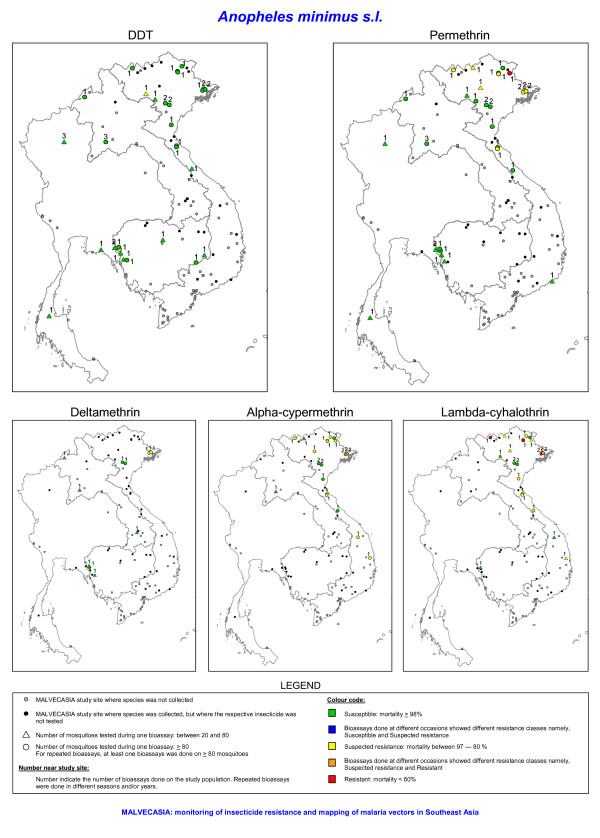
**The insecticide resistance status as defined by the mortality rate obtained with bioassays of *Anopheles minimus s.l*. for five insecticides**. The number of tested mosquitoes has been corrected for morphological misidentifications.

**Figure 5:**
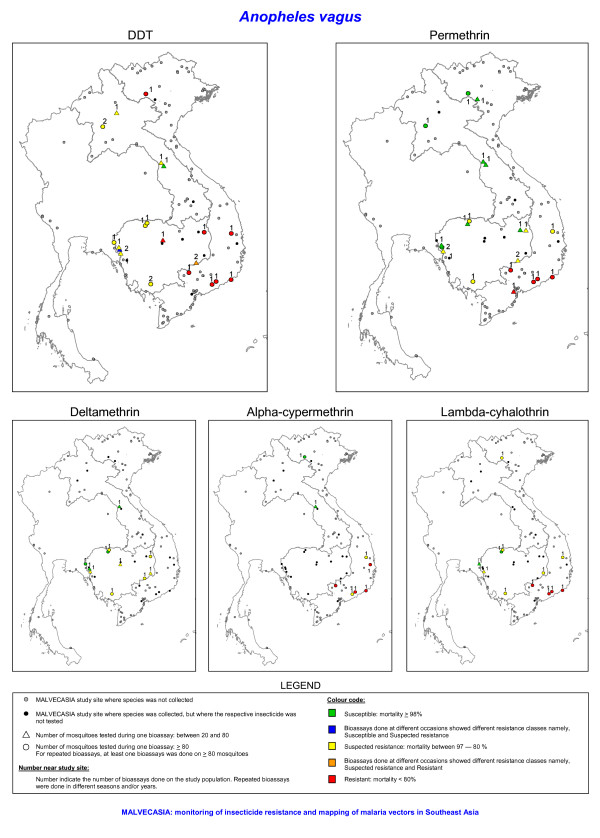
**The insecticide resistance status as defined by the mortality rate obtained with bioassays of *Anopheles vagus *for five insecticides**. The number of tested mosquitoes has been corrected for morphological misidentifications.

Out of 122 prospected sites (Figure 1), 102 sites were positive for at least one of the four study species. It was not always possible to collect the recommended number (i.e. ≥ 80 specimens per species) of mosquitoes for the bioassay test due to the sometimes low density of the test species in the villages. Nevertheless, tests done on 20 to 80 mosquitoes were included in the results but need to be considered as indicative for the resistance status. These results are shown in a different way than the results from test done on 80 or more mosquitoes (Figures 2, 3, 4, 5). Bioassay test done on less than 20 mosquitoes were excluded. Moreover if the control mortality was too high tests were excluded as well, hence results are available for 86 sites.

**Figure 6:**
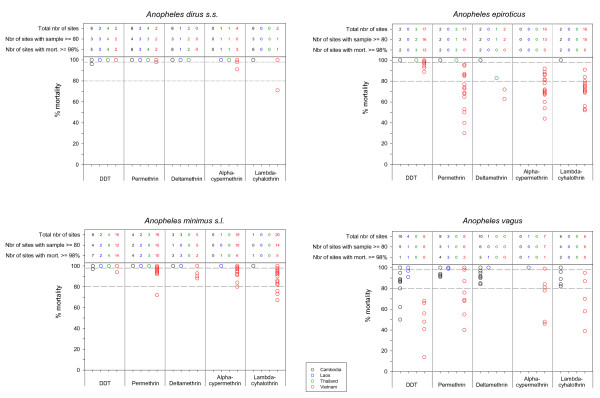
**Overview of the bioassays mortality rates for *Anopheles dirus s.s., An. epiroticus, An. minimus s.l*. and *An. vagus***. Each point represents at least one study site. If more bioassays were done in the same site, only the highest value is plotted. Dotted lines indicate the limits of the WHO resistance classes, i.e. upper limit 98% and lower limit 80% mortality. Colour code black: Cambodia, blue: Laos, green: Thailand, red: Vietnam.

**Figure 7:**
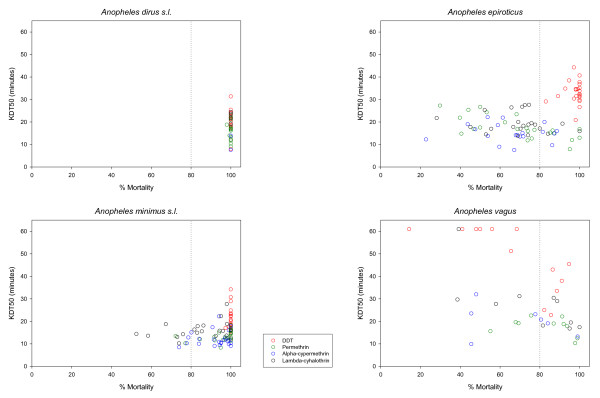
**The time for 50% knockdown (KDT50) in function of the observed mortality for four different insecticides**. Only tests were included when 80 or more mosquitoes were tested and when the morphological identification was reliable (90% of more correct identifications). The resistance cut off value of 80% mortality is indicated by a dotted line.

*Anopheles dirus s.s*. (previously *An. dirus *A [20]) was the only species of the *An. dirus *complex found, except in TKNA. In total 16 *An. dirus s.s*. populations throughout the Mekong region were tested for permethin resistance. In 11 of the 16 sites tests were based on ≥ 80 mosquitoes. All populations showed that this species was susceptible to permethrin. In one site in Cambodia, suspected DDT resistance was found, but this was only based on 23 specimens tested. In central Vietnam, one *An. dirus s.s*. population showed possible resistance to alpha-cypermethrin, tested on 76 specimens. Possible resistance to alpha-cypermethrin in central Vietnam might be more widespread as similar findings were observed in four other sites of central Vietnam (VDAA, VDGA, VGLA and VKHA). These tests were, however, not included in Figures 2 and 6 because the number of mosquitoes used in the control was too low. One *An. dirus s.s*. population was resistant against lambda-cyhalothrin, with a mortality of 75% tested on 66 specimens (Figures 2 and 6). The exposure time to obtain 50% knockdown (KDT50) ranged from 8 till 31 minutes for DDT and from 8 till 24 minutes for pyrethroids (Figure 7). In Western Thailand, TKNA village, three species of the *An. dirus *complex were found namely, *An. dirus s.s*., *Anopheles baimai *(previously *An. dirus *D [20]) and *Anopheles scanloni *(previously *An. dirus *C [20]). The latter species was the most abundant one in this study site. No DDT and permethrin resistance was found in this population.

In the Mekong delta, *An. epiroticus *was highly resistant to all pyrethroid insecticides tested, with most of the population showing mortality lower than 80% (Figures 3 and 6). The results were confirmed for nine sites (VBLA, VBLB, VBTA, VCMC, VCMD, VHCA, VKGS, VSTA and VTVA) by bioassays done on adults reared from wild collected larvae. In Thailand, the two test populations were susceptible to DDT and permethrin. The results on permethrin are only indicative since less than 80 (TPGA: 2004, n = 22 and 2005, n = 70; TRYA: 2003, n = 44 and 2004, n = 57) mosquitoes were tested (Figure 3). The three study populations from Cambodia remained susceptible to permethrin, however one population in Cambodia showed possible deltamethrin resistance. Two populations, VBLA and VBLB, were additionally tested with the discriminating concentrations of etofenprox (0.5%) and cyflutrin (0.15%) [12]. *Anopheles epiroticus *from these two sites showed high levels of resistance (mortality ≤ 57%) against those two insecticides. Thirteen out of the 17 *An. epiroticus *populations were susceptible to DDT, whereas suspected DDT resistance was only observed around Ho Chi Minh City (Figure 3). The KDT50 for pyrethroids varied between 7 and 28 minutes but no relation was observed with mortality (Figure 7), for DDT KDT50 ranged from 21 to 44 minutes.

In Vietnam, 10 out of 19 *An. minimus s.l*. populations were susceptible for permethrin, whereas no resistance was found in Cambodia, Laos and Thailand. In these three countries only a limited number of populations were tested often on a small number of mosquitoes (between 20–80 mosquitoes) (Figures 4 and 6). A similar pattern was observed for alpha-cypermethrin and lambda-cyhalothrin (Figures 4 and 6). Bioassays done on adults reared from wild collected larvae originating from VBKA, VCBB, VLSA, VQNB, VTHA showed even higher resistance for alpha-cypermethrin and lambda-cyhalothrin. For these insecticides almost no information was collected in Cambodia, Laos and Thailand. In Vietnam, six *An. minimus s.l*. populations (VBKA, VBKB, VHBA, VHBB, VLSA and VQNB) were additionally tested with etofenprox 0.5%. The VBKA, VHBB and VQNB populations were susceptible, whereas the populations from the three other study sites showed possible resistance to etofenprox (mortality 95%). Overall the resistance level in *An. minimus s.l*. was less important than the one observed in *An. epiroticus *(Figure 6). Two *An. minimus s.l*. populations showed DDT tolerance, one in western Cambodia and one in northern Vietnam (test on 73 mosquitoes). The KDT50 values for pyrethroids of most *An. minimus s.l*. populations were below 20 minutes (Figure 7).

**Figure 8:**
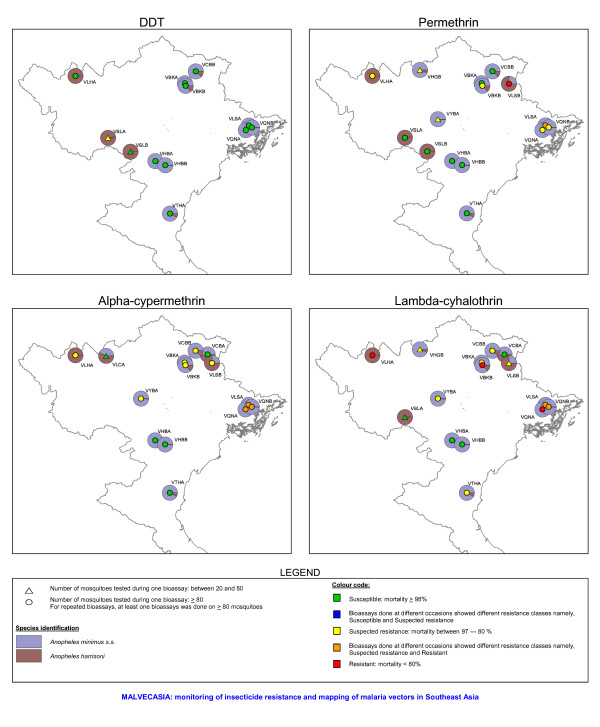
Insecticide resistance status of *Anopheles minimus s.l*. and the distribution of the two members of the *An. minimus *complex, *An. minimus s.s*. and *An. harrisoni *in northern Vietnam.

In Cambodia, southern Laos and Thailand only *An. minimus s.s*. (previous *An. minimus *A [21]) was found in the study sites. Both members of the *An. minimus *species complex, *An. minimus s.s*. and *Anopheles harrisoni *(previous *An. minimus *C [22]), were found in northern Laos and northern Vietnam. Allopatric populations of *An. minimus s.s*. and *An. harrisoni *were found both susceptible and resistant for different pyrethroids (Figure 8). In sympatric populations, resistant mosquitoes were observed within both species. In these sites, the distribution of *An. minimus s.s*. and *An. harrisoni *among dead and alive mosquitoes found in bioassays with permethrin, alpha-cypermethrin and lambda-cyhalothrin was not significantly different (Pooling of the sites by insecticide; Fisher exact test: permethrin, p = 0.852; alpha-cypermethrin, p = 1.000; lambda-cyhalothrin, p = 0.156).

Bioassays were performed on *An. vagus *populations from Vietnam, Laos and Cambodia, whereas this species was not tested in Thailand. Only two out of 20 populations tested with DDT were found susceptible. In Vietnam the level of DDT resistance was very high, with mortality ranging from 68% to 14% (Figures 5 and 6). Permethrin resistance was found in south-central Vietnam. In Cambodia the bioassays indicate possible permethrin resistance, but most tests were done on less than 80 mosquitoes (Figure 5). In populations showing high DDT resistance the 50% knockdown time exceeded 60 minutes (Figure 7).

## Discussion

A cross-country survey of insecticide resistance in *Anopheles *species was set-up in the Mekong region in order to obtain an updated view of the resistant status of the main malaria vectors and to acquire comparable baseline data on insecticide resistance in the region. This cross-country approach is indispensable as malaria transmission in the Mekong region is concentrated in forested areas along the country borders and that decisions on insecticide use should ideally be made at regional level.

Consequently, bioassays were done in almost 90 sites in four countries of the Mekong region applying a standard protocol based on the WHO bioassay test [12]. WHO recommends using non-blood fed, two to five days old, adult female mosquitoes to assess the susceptibility status of a natural *Anopheles *population. This can only be obtained by collecting larvae and rear them to adults. Collecting an appropriate number of larvae of the important vector species was problematic due to the scattered nature of their breeding sites. Therefore, bioassays during this study were done on mosquitoes collected as adults using different collection methods. Since the age effect could not be controlled for, the estimated resistance is likely to be underestimated as shown by Lines and Nassor [23], and Hodjati and Curtis [24]. This was confirmed in the present study when comparing the results obtained from the bioassays on reared and collected adults of *An. minimus s.l*. However, the bioassays results of *An. epiroticus *based on adults reared from larvae and on mosquitoes caught by adult collection methods were generally in agreement. Next to insecticides applied by the national malaria control programmes, two insecticides (DDT and permethrin) were tested in all countries which will serve as common basis for comparison and resistance management. For all insecticides the discriminating concentration as defined by WHO [12] was used.

In the Mekong region, a large number of *Anopheles *species can be found in the vicinity of human dwellings [25]. The identification of these species in the field is often cumbersome due to overlapping morphological characters. Moreover, the vector taxa *An. dirus s.l*. and *An. minimus s.l*. are species complexes of which the members can only be distinguished by molecular means. Molecular tests were, therefore, applied in order to identify the members of the different species complexes and to verify the quality of the morphological identification. One of the problems encountered during the monitoring was the difference in morphological identification skills among the field teams which resulted in a loss of information. However, the misidentification did not influence the interpretation of the bioassays as WHO resistance classes were not different when calculations were based on morphological identification alone or when identification error rates were taken into account. Therefore bioassay results were presented on the basis of the morphological identification, but the number tested (i.e. 20–80 mosquitoes 80 or more) was changed according to the misidentification rate.

During the three years survey large differences in insecticide resistance status were observed among species and countries. In Laos, Cambodia and Thailand, insecticide resistance in the malaria vectors, *An. dirus s.s., An. epiroticus *and *An. minimus s.l*., was almost absent. DDT resistance was only found in *An. vagus *although possible DDT resistance has previously been detected in *An. dirus s.l*. and *An. minimus s.l*. from northern Thailand [11,26]. In the Mekong delta, *An. epiroticus *was highly resistant to all pyrethroid insecticides tested. In 1996–1997, permethrin resistance has been observed in this species in Vietnam [9], but the observation was limited to one study site. A limited number of pyrethroid resistant *An. minimus s.l*. populations were found in northern Vietnam. Yet, most of the study populations showed possible pyrethroid resistance. *Anopheles dirus s.s*., the main vector in forested malaria foci, was susceptible to permethrin. Though, in central Vietnam, it showed possible resistance to the type II pyrethroids alpha-cypermethrin and lambda-cyhalothrin.

This study did not seek to understand the spatial and species related differences in susceptibility status. These patterns are generated by a complex interaction between the population biology and genetics of the vector and the insecticide pressure presence in the ecosystem. The relative role of insecticide pressure from agriculture and vector control in the selection of insecticide resistance is difficult to assess. Historically, DDT has been used to control malaria in the Mekong region. Nowadays all countries use pyrethroid based vector control measures such as ITNs to contain malaria and up scaling of treated nets is going on in high transmission areas. Pressure from agricultural activities is likely to show large spatial variation given the differences in land use in the Mekong region. The almost absence of insecticide resistance in *An. dirus *might be explained by the fact that this species is confined to natural forest environments [27] with very low insecticide pressure from agriculture. The high coverage in endemic foci of ITN, where *An. dirus *is the main vector, seems not to have selected insecticide resistance. *Anopheles epiroticus*, highly resistant to pyrethroids, breeds in the Mekong delta [28] where intense agriculture activities are deployed but where the use of vector control is limited due to the very low malaria endemicity. *Anopheles epiroticus *is related to shrimp farming [29]. In the rice/shrimp system farmers use less pesticide compared to the rice production alone [30], hence the high pyrethroid resistance in *An. epiroticus *is not easily explained. However, the Mekong delta accumulates pollutants from different sources making exposure of *An. epiroticus *larvae to pesticides likely. Likewise, differences in breeding ecology might explain the different resistance status observed among species occurring in the same site, as for example *An. dirus s.s., An. minimus s.s*. and *An. vagus *in eastern Cambodia. The high DDT resistance observed in different *An. vagus *populations is puzzling and might indicate that DDT pressure is still available despite the fact that DDT is not used any more for the control of malaria. The patchy distribution of insecticide resistance of *An. minimus s.l*. in northern Vietnam is remarkable. These spatial differences are not due to geographical distribution of the sibling species, *An. minimus s.s*. and *An. harrisoni *with different insecticide susceptibility status, but are likely to be due to differences in insecticide pressure in the different sites.

Insecticide resistance was only observed in low or transmission free areas of Vietnam [5,29,31], hence an immediate change of malaria vector control strategy is not required. However, the current malaria situation in this area is a consequence of the implementation of the comprehensive national malaria control programme including pyrethroid treated bed net distribution and targeted indoor spraying [5,10,32]. Furthermore, access to effective preventive measures is essential to further contain malaria in endemic foci and vector control can play a role in restraining the spread of drug resistance by decreasing the size of the parasite population and the circulation of the resistant parasites in the population [6]. Consequently, the spread of insecticide resistance from low transmission areas to endemic foci should be carefully monitored and if possible prevented. In this context resistance management is important especially for *An. minimus s.l*. which is present in malaria endemic and non-endemic areas.

Effective resistance management depends on early detection and monitoring of trends in resistance status, understanding the underlying mechanisms, and assessing the operational implications of the observed insecticide resistance, so that rational insecticide choice can be made [7]. The operational implications will largely depend on the resistance mechanisms involved as shown by Chandre *et al *[33], Hargreaves et al [34] and Etang *et al *[35]. The almost absence of DDT resistance in *An. minimus s.l*. and *An. epiroticus *indicates that knockdown resistance is unlikely and that other mechanisms could be involved. The observed variation in KDT50 for DDT and pyrethroids is comparable with the variation found for susceptible reference strains of *An. gambiae *[36,37]. In *An. vagus *knockdown resistance is expected based on the observed DDT and pyrethroid resistance and the high KDT50 values. Furthermore, assessing the susceptibility status to organophosphates and carbamate insecticides will complement these data and will provide essential information on possibility of insecticide rotation or mosaic treatment using compounds with different mode of action. This clearly applies to indoor spraying but the possibility of treating an individual net with different families of insecticides has been considered [38].

## Conclusion

This is the first cross-country survey of insecticide resistance of malaria vector species in the Mekong region. After three years of intense insecticide resistance monitoring a clear picture of insecticide resistance status of malaria vectors was achieved. In Laos, Cambodia and Thailand, insecticide resistance in the malaria vectors *An. dirus s.s., An. epiroticus *and *An. minimus s.l*. was almost absent. In Vietnam, insecticide resistance was mainly observed in low or transmission free areas and there is no need to actually change the malaria control strategy currently implemented in Vietnam. Though, resistance management is important because the risk of migration of mosquitoes carrying resistance genes from non-endemic to endemic areas. Moreover, trends in resistance status should be carefully monitored, mainly in malaria endemic foci, and the impact of existing vector control tools on resistant populations should be assessed. A unique baseline data on insecticide resistance is now available in the Mekong region, which enable to follow trends in susceptibility status in the region and which will serve as basis for further resistance management.

## Authors' contributions

WVB and MC designed the study; revised and supervised the work critically at all stages and drafted the manuscript. WVB carried out the data analysis. HDT, LKT, TS, DS CS, VB, KK and SP facilitated and carried out the field work. LD, PR and KV carried out the molecular identification of the collected mosquitoes and critically reviewed the manuscript. VO developed the Southeast Asian-GIS (SEAGIS) used to map the data.
